# Capacity mapping of national ethics committees in the Eastern Mediterranean Region

**DOI:** 10.1186/1472-6939-10-8

**Published:** 2009-07-04

**Authors:** Alaa Abou-Zeid, Mohammad Afzal, Henry J Silverman

**Affiliations:** 1 Research Policy and Cooperation Unit, Eastern Mediterranean Regional Office of the World Health Organization, Cairo, Egypt; 2 University of Maryland School of Medicine, Baltimore, Maryland, USA

## Abstract

**Background:**

Ethics issues in the areas of science, technology and medicine have emerged during the last few decades. Many countries have responded by establishing ethics committees at the national level. Identification of National Ethics Committees (NECs) in the Eastern Mediterranean (EM) region and the extent of their functions and capacity would be helpful in developing capacity building programs that address the needs of these committees. Accordingly, we conducted a survey to determine the characteristics of existing NECs in the EM region.

**Methods:**

We developed a questionnaire to collect information on different aspects of NECs. The questionnaire was sent to the WHO country office in each of the 22 Member States in the EM region. We used descriptive statistics to analyze the data.

**Results:**

We obtained responses from 77% (17/22) of the EM countries; 88% (15/17) of the countries stated they had NECs. Of these NECs, 40% (6/15) were involved in the ethics of science and technology, 73% (11/15) in medical ethics, and 93% (14/15) in medical research ethics; 10 NECs stated they reviewed research protocols. Of the respondent NECs, 25% (4/15) met at least on a monthly basis. Regarding training, 21% of the members from all of the NECs had received formal training in ethics; 53% (8/15) of the NECs had none of their members with formal training in ethics. Regarding support, 33% (5/15) received financial support and 60% (9/15) had administrative support.

**Conclusion:**

While many countries in the EM region report the existence of NECs, many meet infrequently, many have members without formal training in ethics, and many lack important financial and administrative resources. Further efforts should be directed towards capacity building programs that include ethics training and provision of important infrastructure resources for these committees.

## Background

Ethics issues in the areas of science and technology, environment, and medicine have become increasingly prominent during the last few decades. In particular, medical ethics issues have surfaced in the areas of assisted reproductive technologies, organ transplants, genetics, newborn screening, and research [1-5]. Furthermore, developing countries have witnessed an intensification of research activities that have not been accompanied by a corresponding increase in research ethics capacity, including functioning ethics review systems to review the ethics of research [6-8]. Insufficient ethics capacity has been demonstrated in the different countries of the Eastern Mediterranean (EM) region [9,10].

An important infrastructure element of ethics capacity at the national level is the establishment of National Ethics Committees (NECs). Such NECs could focus on providing guidance and advice to policy makers, developing normative instruments in ethics, establishing training programs in ethics, and reviewing research protocols, either those to be conducted in the country or only those that are international in scope [11,12]. Identification of the existing NECs in the EM region and the extent of their functions and capacity would be helpful in targeting areas for improvement and providing capacity building programs to meet the needs of these NECs. Such information would help guide organizations such as the Eastern Mediterranean Regional Office (EMRO) of the WHO and the United Nations Educational, Scientific, and Cultural Organization (UNESCO) in their support to the development, promotion, and strengthening of such committees. Accordingly, this survey study was undertaken to determine the characteristics of existing NECs in the Member States in the EM region.

## Methods

### Setting

We conducted a survey involving the 22 Member States of the EM region, which includes: Afghanistan, Bahrain, Djibouti, Egypt, Islamic Republic of Iran, Iraq, Jordan, Kuwait, Lebanon, Libyan Arab Jamahiriya, Morocco, Oman, Pakistan, Palestine, Qatar, Saudi Arabia, Somalia, Sudan, Syrian Arab Republic, Tunisia, United Arab Emirates, and Yemen.

### Survey Instrument

We developed a structured questionnaire to identify the following characteristics of NECs: meeting frequency, member composition, main activities of the committee, its role in the review of research, challenges to its functions, self-rated capacity to perform their activities, extent of training in ethics of its members, the existence of country laws regulating research, the presence of national ethics guidelines, and the existence of financial and material resources.

### Process

The survey instrument was sent to the WHO country office in each country. Subsequently, the WHO Country Office delivered the survey instrument to an individual involved with the existing NEC or the person involved in ethics within the Ministry of Health. A cover letter explained the purpose of the survey, type of information being collected, the voluntary nature of the survey, contact information of who can answer questions about the survey, and assurance that while aggregate data would be made publically available, specific data of individual NECs would remain confidential. Reminders were sent by EMRO/WHO to the WHO Country Offices to enhance the response rate of collection. Completed questionnaires were returned to EMRO/WHO where the questionnaires were checked for completeness. Subsequently, the data was sent to the University of Maryland, School of Medicine for data management and statistical analysis. This process took place between February and May, 2007.

### Analysis

We used descriptive statistics to analyze the data obtained from the surveys.

### Ethical Aspects

The Institutional Review Board at the University of Maryland, Baltimore, USA, reviewed this study. Informed consent was obtained by participants who completed the survey instrument.

## Results

We obtained responses from 77% (17/22) of the EM countries. Of the 17 countries that responded, 88% (15/17) confirmed the existence of an NEC (Table 1). It appears that several of these NECs are mainly functioning as committees that review research rather than as a national ethics committee dealing with general ethics issues. For example, Afghanistan mentioned that it has an Institutional Review Board, Sudan stated it has a Sudan National Ethical Review Committee and Yemen mentioned it has The National Ethics Committee of Medical and Health Research.

**Table 1:** Responses of Countries Regarding the Presence of a National Ethics Committee

**Country Names**	**Presence of a National Ethics Committee**
Afghanistan, Bahrain, Egypt, Islamic Republic of Iran, Jordan, Kingdom of Saudi Arabia, Lebanon, Libya, Oman, Pakistan, Syrian Arab Republic, Sudan, Tunisia, United Arab Emirates, and Yemen.	Yes

Djibouti, Morocco	No

Iraq, Palestine, Somalia, Qatar, Kuwait	Did Not Respond

### Activities

Regarding frequency of meetings, 4 of the 15 NECs met at least on a monthly basis (one met twice a month and three met every month), whereas the other NECs met less frequently. Of the existing NECs, 40% (6/15) were involved with the ethics of science and technology, 73% (11/15) in medical ethics, and 93% (14/15) in medical research ethics. None of the NECs were involved in environmental ethics.

The specific activities of the NECs are listed in Table 2. Of the 14 NECs involved in medical research, ten reviewed research protocols. Of these ten NECs, 8 reviewed international collaborative research, 9 reviewed national research, and 7 reviewed institutional research. Regarding the documents used to review research, 7 used the Declaration of Helsinki, 6 used the CIOMS guidelines, 6 used national guidelines, and 4 reported using the Belmont Report. Of the 7 NECs that gave responses, the number of protocols reviewed at meetinge ranged from 1 to 8.

**Table 2:** Activities of National Ethics Committees (NECs)

**Activities**	**Numbers of NECs performing activity (%)** **(n = 15)**
Review of medical research protocols	67% (10/15)
Conduct training activities at the national level	47% (7/15)
Develop publications in ethics	67% (10/15)
Provide an advisory role to policy makers	47% (7/15)
Provide "overseeing" "follow up" to policy makers on ethics issues and decisions	53% (8/15)

### Composition

Table 3 reveals the overall reported composition of the NECs. As shown, membership on the NECs reflected broad representation from governmental, medical, and public areas. Forty-seven percent (7/15) of the NECs had members from five or more different specialties. The number of members on the NECs ranged from 2 to 24 and 14 of the NECs had at least 6 members on the committee. The average membership was 12 with a median of 11.

**Table 3:** Composition of existing National Ethics Committees (NECs) countries that reported its existence (n = 15)

**Specialty**	**Numbers of NECs with member specialty (%)**
National Ministry	40% (6/15)
Medical Doctor	100% (15/15)
Scientist	33% (5/15)
Social Scientist	27% (4/15)
Public Health	13% (2/15)
Epidemiologist	13% (2/15)
Nurse	20% (3/15)
Pharmacy	13% (2/15)
Legal Expert	60% (9/15)
Religious	33% (5/15)
Community Member	13% (2/15)
Journalist	13% (2/15)
Bioethicist	13% (2/15)
Human Rights Council	13% (2/15)
Other	53% (8/15)

### Challenges

NECs were asked to self-rate their capacity to perform their duties; 7% (1/15) rated its capacity as being "excellent", 47% (7/15) rated their capacity as being "good", 27% (4/15) rated their capacity as "moderate", and 14% (2/15) rated its capacity as being "limited"; one NBC gave no answer. Table 4 shows the issues that were perceived as challenges to the NECs' capacity to perform it duties.

**Table 4:** Challenges to the capacity of National Ethics Committees to perform its duties (n = 15)

**Challenges**	**Numbers of RECs reporting challenge (%)**
The need to develop appropriate national ethical guidelines	86% (13/15)
Lack of training for members in medical ethics	67% (10/15)
Lack of ongoing training in medical ethics	73% (11/15)
Inadequate ability to monitor approved protocols	60% (9/15)

### Training

Regarding training, 21% of the members from all of the NECs indicated they had received formal training in ethics, while 53% (8/15) of the NECs had none of their members with formal training in ethics. Table 5 shows the topics in research ethics that were rated as being important by more than 50% of the NECs.

**Table 5:** Topics reported as being "very important" or "quite important" for the National Ethics Committees (NECs).

**Topics**	**Numbers of NECs reporting topic (%)**
Monitoring and oversight	87% (13/15)
Assessment of understanding of informed consent	80% (12/15)
Privacy and confidentiality	80% (12/15)
Provision of appropriate risk reduction measures	80% (12/15)
Assessment of cultural sensitivity for informed consent	73% (11/15)
Placebo controlled trials	73% (11/15)
Determination of appropriate subject selection in vulnerable populations	67% (10/15)
Assessment of anticipated benefits	67% (10/15)
Community participation	67% (10/15)
Determinations to conduct Phase I, II, and III clinical trials in a country or community	60% (9/15)
Incentives for participation	60% (9/15)

### Laws Regulating Research

Table 6 shows the data regarding the existence of country laws that govern research. Sixty percent (9/15) of the NECs mentioned that it was possible to conduct medical research without any ethics approval. Regarding challenges to the use of research ethics guidelines, 86% (13/15) of the NECs included the "variable use of ethical guidelines across to local ethics committees within their country"; 80% (13/15) mentioned the "lack of sensitivity to local socio-political-economic-cultural context"; and 53% (8/15) stated the "difficulties adapting international guidelines to local condition".

**Table 6:** Laws regulating research existing within the countries of the National Ethics Committees (NECs)

**Types of Laws Regulating Research**	**Numbers of NECs reporting type of law (%)**
Governing ethical review of research	46% (7/15)
Protection of research subjects	46% (7/15)
Requirement of informed consent	67% (10/15)
Establishment of research ethics committees	40% (6/15)

### Financial and Material Resources

Regarding financial support, 33% (5/15) of the NECs stated they received financial support and all of these NECs mentioned that such support came from the national government. Two of the NECs stated that members received financial compensation for their activities. Regarding office space, computer equipment, and administrative support, 73% (11/15), 67% (10/15), and 60% (9/15) of the NECs, respectively, indicated that such resources were available to their committees.

## Discussion

The major finding of this capacity mapping is that many of the countries in the Eastern Mediterranean Region have NECs and many of these are engaged predominantly in the areas of medical ethics and research ethics. Activities include conduct of training at the national level, publishing in ethics, providing an advisory role to policy makers and the review of research protocols. The strong focus on research ethics is significant for several reasons. First, there has been a recent intensity of research activities in the EM region coupled with the need to have strong ethics review systems. Second, there is a concern with the potential for exploitation in the area of international research, considering the presence of extreme poverty and lack of access to health care among a high proportion of the populations in the EMRO countries [7,12]. In addition to performing review of research protocols, many of the NECs stated it conduct training in ethics. One activity not mentioned by the NECs was performing oversight of local ethics committees. Coleman and Boueseau has recently written on the need to have mechanisms to monitor outcomes assessment of research ethics committees, which would lead to enhanced quality of ethics review [13].

Many of the NECs have a diverse membership (including members from the lay public) with most consisting of more than six members. However, our results indicate that many of these NECs lack the essential human, financial, and capital resources needed for a well-functioning committee. For example, only 21% of all of the members indicated having received some formal training in ethics and 8 NECs reported having no member with such formal ethics training. Many of the respondent NECs stated they operate without financial and secretarial support. Finally, only about 25% of the NECs met on at least a monthly basis.

NECs stated that challenges to effective functioning included the lack of national ethics guidelines and the lack of training for it members in medical ethics. Challenges in the area of research ethics included variable use of ethical guidelines across local ethics committees within the country; lack of sensitivity of guidelines to the local context; and difficulties adapting international guidelines to local conditions. Indeed, despite the existence of laws regulating research in several of the countries, many NECs mentioned that it was possible to conduct research without ethics review. This information is consistent with previous studies demonstrating that many investigators fail to obtain ethics review of their submitted proposals to sponsoring agencies [6,9,10]. Further efforts are needed to ensure compliance with existing laws in addition to the development of national regulations in countries that lack such regulations. These challenges affirm the widespread concern that despite the existence of international research ethics standards, guidelines need to be developed that are sensitive and relevant to the local context of countries in the EM Region [6,12,14].

Our study adds to the scarce literature that exists on national ethics committees. Recently, Fischer published his narrative findings regarding National Bioethics Committees in selected States of North African and the Middle East [15]. Also, Kirigia and colleagues assessed the status of national research bioethics committees in the 46 Member States in the WHO Africa Region [16]. Their study had a 61% response rate and 64% (18/28) of the countries that responded confirmed the existence of a national research ethics committee dealing with bioethics research issues. The authors found that the NECs lacked ethicists and qualified laypersons on the committees and, similar to our finding, only a minority of the respondent ethics committees met on a monthly basis (44%). No data were obtained regarding ethics training of the members and the extent of financial and material resources available to the NECs.

There are several limitations to our study. First, although the survey instrument was sent to the WHO Country office, we are not entirely sure of who completed the survey and whether the person was qualified to report on the NECs' functions and capacity. We also do not know whether the data sent to EMRO was discussed among members of the individual NECs. Second, regardless of who completed the survey, it was based on a process of self-report and accordingly, there might have been a tendency to over report the achievements of individual NECs, as well as underreport weaknesses. Lack of knowledge of the specific process with which these surveys were completed call into question the exact validity of the responses. Another limitation of our survey was that we failed to inquire about other types of information. For example, we neglected to inquire about the gender composition of the individual NECs. Also, we did not ask about the legal mechanism that allowed for the existence of the NECs, for example, by law, ministerial decrees, or by decree of the prime minister. Finally, the survey asked for the "sector with which each member represents", which generated some vague answers. For example, many responses stated 'community member' without further details as to the type of person represented by such a label.

Despite these limitations, we did identify several areas of needs for these committees from our survey. For example, the documentation of the need for ethics training should further motivate several organizations to continue with their existing strategic initiatives to enhance ethics capacity in the EM and Arab Regions [17]. UNESCO has focused its ethics capacity-building activities on the development and enhancement of national bioethics committees, which can be effective platforms from which to implement their several declarations in bioethics: the Universal Declaration on the Human Genome (1997), the International Declaration on Human Genetic Data (2003), and the Universal Declaration on Bioethics and Human Rights (2005) [18]. UNESCO also has an Assisting Bioethics Committees program that provides national bioethics committees with practical information and technical support [19]. In terms of ethics training, UNESCO's Ethics Education Programme has organized several conferences on ethics and research ethics in the Arab Regions [20].

The Eastern Mediterranean Regional Office (EMRO), the World Health Organization's regional office in Cairo, Egypt, has also contributed to the enhancement of ethics capacity in the EM region [21]. EMRO has also provided funding for individuals to receive intensive training in bioethics and has provided support for regional workshops and conferences in research ethics.

The Fogarty International Center of the National Institutes of Health provides grants to establish intensive training programs to enhance the bioethics career development of individuals from resource-limited countries [22]. The University of Maryland, for example, is currently providing a Certificate Program in Research Ethics for individuals from the Middle East and the Aga Khan University enables individuals to undertake Masters level training in bioethics [23,24]. Finally, the Welcome Trust in the United Kingdom provides support to build ethics capacity in resource-poor countries that have well-established research centers. Funding is provided for project grants and for seminars and other capacity building initiatives [25].

This study also makes clear the following recommendations for NECs in the EM region that could enhance their functioning: a) NECs should strive to meet regularly (e.g., at least once a month); b) member composition should be diverse, be gender balanced, and have the ethics expertise to ensure the performance of its mandated functions; c) NECs should recommend appropriate policies and legislations to guide, promote, and provide oversight to research ethics review systems; d) efforts should be directed toward the institutionalization of training in ethics and human rights at all stages of education and training of all health professionals; and e) innovative mechanisms should be established for financing NEC activities and reducing logistical barriers that impede regular meetings.

## Conclusion

The role of national ethics committees is instrumental in forging the enhancement of ethics capacity in their respective countries. Such committees can be helpful in formulating ethics guidelines, advising governmental officials in formulating policy, enhancing awareness of controversial ethics issues, and promoting the review of research both at the national and institutional level. This survey has identified the existing NECs in the EM Region and areas that need to be further addressed to enhance their functioning.

## Competing interests

The authors declare that they have no competing interests.

## Authors' contributions

AAZ conceived the idea of the survey, helped with the development of the survey, reviewed the analysis of the aggregate data, and helped with the writing of the manuscript. MA helped conceive the idea of the study and reviewed the final draft of the paper. HJS helped develop the survey tool, analyzed the aggregate data, and helped with the writing of the manuscript. All authors read and approved the final manuscript

## Pre-publication history

The pre-publication history for this paper can be accessed here:


